# Supplementary material for: A direct comparison of patient-reported outcomes and experiences in alternative models of maternity care in Queensland, Australia
Source: PLoS One. 2022 Jul 12;17(7):e0271105. doi: 10.1371/journal.pone.0271105 (PMC9275696; doi:10.1371/journal.pone.0271105)
Supplement: S1 Table — (DOCX) [file pone.0271105.s001.docx]

**S1 Table**: **Assessment of outcomes and potential confounders**.

| **Variable** | **Measurement** | **Categories/Scale** |
| --- | --- | --- |
| **Maternal Socio-demographic Characteristics** | |  |
| Maternal Age at Birth | Calculated from maternal date of birth and baby’s date of birth. | Less than 25yrs, 25-29yrs, 30-34yrs, 35yrs or older |
| Pre-pregnancy BMI | Calculated as weight (kgs)/height(m)^2^ from reported pre-pregnancy weight and height. BMI was converted to four categories: underweight (BMI <18.50), normal weight (BMI from 18.50 to 24.99), overweight (BMI from 25.00 to 29.99), obese (BMI ≥30.00). Consistent with previous methods [1–3], measures of BMI less than 15 and greater than 50 were considered extreme outliers and excluded (reported as missing). Of the total sample, 6.8% of women had missing data for BMI (either due to nonresponse or extreme outliers) and were retained as a variable level coded as *‘Missing’*. | Underweight (<18.5), Normal weight (18.5-24.99), Overweight (25-29.99), Obese (≥30), Missing |
| Area of Residence | Derived from suburb/town and postcode of women’s usual place of residence at the time her baby was born. Categories were created based on the Australian Standard Geographical Classification system [4]. Women who resided outside of Queensland at the time of index birth were excluded from all analyses. | Major city, Inner regional, Outer regional, Remote |
| Education | ‘*What is the highest level of schooling you have completed?*’ ‘Grade 10 or equivalent or less’ if indicated *‘no formal qualifications’* or *‘year 10 or equivalent’*; ‘Grade 12 or equivalent’ if indicated *‘Year 12 or equivalent (e.g., Higher School Certificate)’*; Vocational education if indicated *‘Trade/apprenticeship’* or *‘Certificate/diploma’*; ‘Tertiary education’ if indicated *‘University degree’* or *‘Higher university degree’*. | Grade 10 or equivalent or less, Grade 12 or equivalent, Vocational education, Tertiary education |
| Aboriginal or Torres Strait Islander Identification | ‘Yes’ if indicated being ‘*Aboriginal but not Torres Strait Islander*’, ‘*Torres Strait Islander but not Aboriginal*’ or ‘*Both Aboriginal and Torres Strait Islander*’. ‘No’ if indicated ‘*Neither Aboriginal or Torres Strait Islander*’. | No, Yes |
| Language Spoken at Home | *‘What languages do you speak at home?’* ‘English’ if indicated *‘English’* only; ‘other language(s) with or without English’ if indicated *‘English’* and *‘other’* or *‘other’* without English. | English, Other language(s) with or without English |
| Country of Birth | *‘Where were you born?’* | Australia, Other country |
| **Maternal Reproductive History and Plurality of Index Pregnancy** | |  |
| Parity | Women were asked how many times they had been pregnant and had given birth. Women who indicated one pregnancy or one birth were coded as ‘Primiparous’. Women indicating two or more births were coded as ‘Multiparous’. | Primiparous, Multiparous |
| Previous Caesarean | Women were asked how many times they had had a caesarean birth (including the index birth). Coded as ‘At least one’ if they were multiparous and indicated two or more caesarean births (any mode of index birth) or one caesarean birth if their index birth was a vaginal birth. ‘None’ if they were primiparous or multiparous and indicated 0 caesarean births or one caesarean birth and the mode of the index birth was a caesarean birth. | None, At least one |
| Birth Plurality | Birth plurality was determined by the type of survey (singleton or multiple) returned by women. | Singleton, Multiple |
| **Complications Arising During Index Pregnancy** | |  |
| Women were asked if they had experienced a range of different complications during their pregnancy: ‘*during pregnancy, did a care provider tell you that…* | |  |
| Depression | *…you were experiencing depression?’* | No, Yes |
| Anxiety | *…you were experiencing anxiety?’* | No, Yes |
| Gestational diabetes | *…you had gestational diabetes?’* | No, Yes |
| Hypertension/pre-eclampsia | *…you had high blood pressure (hyptension, pre-eclampsia)?’* | No, Yes |
| Placenta praevia | *…you had placenta praevia (placenta close to or covering your cervix)?’* | No, Yes |
| Amount of amniotic fluid was a concern | *…your amount of amniotic fluid (‘waters’) was a concern?’* | No, Yes |
| Problem with cervix | *…that you had a problem with your cervix?’* | No, Yes |
| A problem with the baby’s cord | *…that there was a problem with your baby’s cord?’* | No, Yes |
| Baby was too big | *…your baby was too big?’* | No, Yes |
| Baby was too small | *…your baby was too small?’* | No, Yes |
| Preterm labour | *…that you were in preterm labour (in labour before you were 37 weeks pregnant)??’* | No, Yes |
| Membranes ruptured in the absence of labour | *…your membranes had ruptured (waters had broken) and labour did not start?’* | No, Yes |
| **Obstetric Intervention and Maternal Health Outcomes** | |  |
| Mode of birth | Women were asked separately how their baby was born and if they had a caesarean birth scheduled in advance. Coded as ‘Unassisted vaginal birth’ if indicated *‘An unassisted vaginal birth (no forceps or vaccum)’*; ‘Assisted vaginal birth’ if indicated *‘A vaginal birth – assisted with a vacuum’, ‘A vaginal birth – assisted with forceps’* or *‘A vaginal birth – assisted by forceps and a vacuum’*; ‘Scheduled caesarean birth’ if indicated *‘A caesarean birth’* and *‘Yes’* to a caesarean birth scheduled in advance; ‘Unscheduled caesarean birth’ if indicated *‘A caesarean birth’* and *‘No’* to a caesarean birth scheduled in advance. Four dichotomous variables were created for each mode of birth: unassisted vaginal, assisted vaginal birth, scheduled caesarean birth, unscheduled caesarean birth. | Unassisted vaginal:  No, Yes  Assisted vaginal birth:  No, Yes  Scheduled caesarean birth:  No, Yes  Unscheduled caesarean birth:  No, Yes |
| Induction of labour | Women were asked if they received a range of interventions to induce labour. Coded as ‘Yes’ if indicated *‘Yes’* to having a membrane sweep, a tablet, pessary, gel or tape inserted into their vagina, a care provider rupture their membranes (break their waters) or a Syntocinon infusion drip ‘*to try to induce (start) labour’,* or indicated any of the above to an open ended item *‘did you have or try anything else to induce (start) labour)?.* ‘No’ for all other responses. | No, Yes |
| Epidural or spinal block for pain relief during labour | Women were asked separately if they had any labour (even if they had a caesarean scheduled in advance) and if they had an epidural or spinal block for pain relief during labour. Coded as ‘Yes’ if they indicated *‘Yes, and it was very helpful*’,*‘Yes, and it was somewhat helpful’* or *‘Yes, but it was not at all helpful’* to having an epidural or spinal block. ‘No’ if indicated *‘No’* to having an epidural or spinal block or *‘No’* to having any labour. | No, Yes |
| Constant fetal monitoring during labour | Women were asked separately if they had any labour (even if they had a caesarean scheduled in advance) and how their baby was monitored (checked) during labour. Women indicating either ‘*A monitor was used constantly with a belt around my stomach*’ or ‘*A monitor was used constantly with a clip on my baby’s head*’ were coded as ‘Yes’. Remaining women were coded as ‘No’ if they indicated ‘*A hand held monitor was used now and then*’, ‘*Staff listened with a stethoscope (or ear trumpet) now and then*’, ‘*A monitor was used now and then, with a belt around my stomach*’ or ‘*My baby was not monitored*’. Open-text responses were back-coded into existing response categories where possible. Women indicating *‘No’* to having any labour were coded as ‘No”. | No, Yes |
| Vaginal examinations during labour | Women were asked separately if they had any labour (even if they had a caesarean scheduled in advance) and how many times did a care provider perform a vaginal (internal) examination after labour had started. Women who indicated *‘No’* to having any labour were coded as 0 vaginal examinations. | Scale |
| Episiotomy | *During your birth, did you have an episiotomy (cut with scissors or a scalpel) to enlarge your vaginal opening?* Coded *‘Yes’* if indicated ‘Yes’. Coded *‘No’* if indicated ‘No’ or ‘Not sure’ or if women had a caesarean birth. | No, Yes |
| Perineal tear | *During your birth, did you have a tear (for example, near the opening of your vagina)?* Coded*‘Yes’* if indicated ‘Yes’. Coded *‘No’* if indicated ‘No’ or ‘Not sure’ or if women had a caesarean birth. | No, Yes |
| Perineal tear following episiotomy | Women who indicated ‘Yes’ to both episiotomy and perineal tear were coded as *‘Yes’*. All other responses or a caesarean birth were coded as *‘No’.* | No, Yes |
| Perineal status | Women were separately asked *‘after birth, did you have stitches near the opening of your vagina?’* Coded as *‘Perineum intact’* if indicated ‘No’ or ‘Not sure’ to both episiotomy and perineal tear or had a caesarean birth; *‘Perineal trauma with no sutures’*  if indicated ‘Yes’ to having an episiotomy or perineal tear and indicated ‘No’ or ‘Not sure’ to having stitches; *‘Sutured perineal trauma’* if indicated ‘Yes’ to having an episiotomy or perineal tear and indicated ‘Yes’ to having stitches. | Perineum intact, Perineal trauma with no sutures, Sutured perineal trauma |
| Maternal length of hospital stay | *‘In total, how many nights did you stay in hospital or birth centre after birth?’* Women who indicated that they did not stay in a hospital or birth centre overnight were coded as 0. | Scale (in nights) |
| Experienced breastfeeding problems | Women were asked if they had experienced breastfeeding problems after their most recent birth (index birth) with response options of ‘Yes’, ‘No’, and ‘Does not apply to me’. Coded *‘Yes’* if indicated ‘Yes’ and coded *‘No’* if indicated ‘No’ or ‘Does not apply to me’. | No, Yes |
| Experienced depression after birth | Women were asked if they had experienced feeling depressed after their most recent birth (index birth) with response options of ‘Yes’, ‘No’, and ‘Does not apply to me’. Coded *‘Yes’* if indicated ‘Yes’ and coded *‘No’* if indicated ‘No’ or ‘Does not apply to me’. | No, Yes |
| Experienced anxiety after birth | Women were asked if they had experienced feeling anxious (worried) after their most recent birth (index birth) with response options of ‘Yes’, ‘No’, and ‘Does not apply to me’. Coded *‘Yes’* if indicated ‘Yes’ and coded *‘No’* if indicated ‘No’ or ‘Does not apply to me’. | No, Yes |
| Diagnosed depression after birth | *Since your birth, have you been told by a health professional that you were experiencing depression?* | No, Yes |
| Diagnosed anxiety after birth | *Since your birth, have you been told by a health professional that you were experiencing anxiety?* | No, Yes |
| Maternal hospital re-admission for reasons related to birth/having a baby | *Have been re-admitted to hospital for your own health since you first came home (or since giving birth to your baby at home)?* Women who indicated ‘Yes’ were given an open-text option to indicate why. Open-text responses were coded into ‘Yes, for reasons directly related to birth/having a baby’, ‘Yes, for reasons indirectly related to birth/having a baby’, ‘Yes, for reasons unrelated to birth/having a baby’, and ‘Yes, for reasons unstated or unclear’. The maternal hospital re-admission for reasons related to birth/having a baby was coded as *‘Yes’* if indicated ‘Yes, for reasons directly related to birth/having a baby’, or ‘Yes, for reasons indirectly related to birth/having a baby’ and *‘No’* for all other responses. | No, Yes |
| **Infant Health Outcomes** | |  |
| Preterm birth (< 37 weeks) | Calculated from reported gestational age at birth. Gestational age under 37 weeks was coded as *‘Yes’* and gestational age equal to or greater than 37 weeks was coded as *‘No’*. | No (≥37 weeks), Yes (<37 weeks) |
| Low birth weight (< 2,500 grams) | Calculated from reported infant weight at birth. Infant weight at birth of under 2,500 grams was coded as *‘Yes’*. Infant weight above equal to or greater than 2,500 grams was coded as *‘No’*. | No (≥2,500 grams), Yes (<2,500 grams) |
| Neonate admission to NICU | *Was your baby ever cared for in a neonatal unit (e.g., special care nursery (SCN) or neonatal intensive care unit (NICU))?* | No, Yes |
| Neonate’s length of stay in NICU | Of the women who indicated ‘Yes’ to neonate admission to NICU: *For how long was your baby in neonatal care in total?* Responses in hours, days, and weeks were converted to total days and coded as *‘Less than 48 hours’, ’48 hours to 7 days’ and ‘Greater than 7 days’*. | < 48 hours, 48 hours to 7 days, > 7 days |
| Infant hospital re-admission | *Since your baby first came home from hospital (or since giving birth to your baby at home) has your baby been re-admitted to hospital?* Women who responded with ‘My baby is still in hospital’ excluded from analyses specific to this outcome. | No, Yes |
| Breastfeeding at 13 weeks | *Is your baby still having breastmilk?* ‘Yes’ responses were further coded against the baby’s date of birth and the date of survey completion to determine infant age at the time of survey completion. ‘No’ responses were coded against a further response to *‘how old was your baby when he/she last had breastmilk’*. Coded as *‘Yes*’ if infant was 13 weeks or older when they last had breastmilk. | No, Yes |
| **Information Provision and Decision Making in Pregnancy, Labour, and Birth** | |  |
| The pros and cons of having and not having medical interventions/ procedures during pregnancy were discussed | *‘Did your maternity care provider(s) discuss with you the pros and cons (benefits and risk) of…*   - *Having and not having ultrasound scans?’* - *Having and not having blood tests during your pregnancy?’*   Each intervention/procedure was represented by a dichotomous variable. | Ultrasound scans:  No, Yes  Blood tests:  No, Yes |
| The pros and cons of having and not having medical interventions/ procedures during labour/birth were discussed | *‘Did your maternity care provider(s) discuss with you the pros and cons (benefits and risk) of…*   - *Having and not having a caesarean?’* - *Being induced or not being induced?’* - *Monitoring and not monitoring your baby during labour?’* - *Having and not have vaginal examinations to check the progress of your labour/birth?’* - *Having and not having an epidural/spinal (injection in your back)?’* - *Having and not having an episiotomy?’* - *Having and not having a drip/injection of Syntocinon to birth your placenta?’*   Each intervention/procedure was represented by a dichotomous variable. Coded *‘Yes’* if indicated ‘Yes, discussed during pregnancy’ or ‘Yes, discussed during labour and birth’ and *‘No’* if indicated ‘No’. | Caesarean birth:  No, Yes  Induction of labour:  No, Yes  Fetal monitoring during labour:  No, Yes  Vaginal examinations:  No, Yes  Epidural:  No, Yes  Episiotomy:  No, Yes  Syntocinon to birth placenta:  No, Yes |
| Women did not give consent to the final decision to have or not have medical interventions/procedures during pregnancy | *‘Who made the final decision to…*   - *Have or not have ultrasound scans?’* - *Have or not have blood tests during your pregnancy?’*   Each intervention/procedure was represented by a dichotomous variable. Coded *‘Consented’* if indicated ‘I made the final decision myself, from all my available options’ or ‘My maternity care provider(s) made the final decision and checked if it was OK with me’ and *‘Did not give consent’* if indicated ‘My maternity care provider(s) made the final decision without checking with me’. | Ultrasound scans:  Consented, Did not consent  Blood tests:  Consented, Did not consent |
| Women did not give consent to the final decision to have or not have medical interventions/procedures during labour/birth | *‘Who made the final decision…*   - *To have or not have a caesarean?’* - *To induce or not induce you?’* - *If/how your baby was monitored during labour?* - *To have or not have vaginal examinations?’* - *To have or not have an epidural/spinal?’* - *To have or not have an episiotomy?’* - *To have to have or not have a Syntocinon drip/injection to birth your placenta?’*   Each intervention/procedure was represented by a dichotomous variable. Coded *‘Consented’* if indicated ‘I made the final decision myself, from all my available options’ or ‘My maternity care provider(s) made the final decision and checked if it was OK with me’ and *‘Did not give consent’* if indicated ‘My maternity care provider(s) made the final decision without checking with me’. ‘Induction of labour’ had greater than 5% missing data and was coded as a three-level variable, retaining *‘missing’* as a level. | Caesarean birth:  Consented, Did not consent  Induction of labour:  Consented, Did not consent, Missing  Fetal monitoring during labour:  Consented, Did not consent  Vaginal examinations:  Consented, Did not consent  Epidural:  Consented, Did not consent  Episiotomy:  Consented, Did not consent  Syntocinon to birth placenta:  Consented, Did not consent |
| **Maternal Experiences During Pregnancy** | |  |
| Able to choose gender of care provider | *‘Could you choose whether your care provider(s) for labour and birth was/were male and female?’* Women indicating *‘Yes’* were coded as ‘Yes’ and all other responses coded as ‘No’. | No, Yes |
| Able to choose mode of birth | Women were asked (in regard to mode of birth) *‘which of the following options were available to you?* Women who indicated ‘A vaginal birth only’, ‘A caesarean birth only’, or ‘Not sure’ were coded as *‘No’*. Coded as *‘Yes’* if indicated ‘Either a vaginal birth or a caesarean birth’. | No, Yes |
| Weeks gestations at time of first pregnancy check-up | *Roughly how many weeks pregnant were you when you first started having check-ups in your pregnancy?* Women who did not have any pregnancy check-ups were excluded from analyses specific to this outcome. | Scale (in weeks) |
| Weeks gestation at time of booking appointment | *Roughly how many weeks pregnant were you at your first maternity care appointment (your ‘booking’ visit) in your planned place of birth?* Women who did not have a booking appointment were excluded from analyses specific to this outcome. | Scale (in weeks) |
| Satisfied with timing of booking appointment | Women were asked if they felt their booking appointment was ‘Too early’, ‘Too late’, or ‘About the right time’. Coded as *‘Satisfied’* if indicated ‘About the right time’ and *‘Not satisfied’* for all other responses. Women who did not have a booking appointment were excluded from analyses specific to this outcome. | Satisfied, Not satisfied |
| Number of pregnancy check-ups | *Roughly how many times in total did you see a midwife or doctor for a check-up during your pregnancy?* Women who indicated that they did not have pregnancy check-ups or did not go to a care provider during pregnancy were coded as 0 check-ups. | Scale |
| Satisfied with number of pregnancy check-ups | Women were asked if they felt the number of pregnancy check-ups they received was ‘Too many’, ‘Too few’, or ‘About the right number’. Coded as *‘Satisfied’* if indicated ‘About the right number’ and *‘Not satisfied’* for all other responses. | Satisfied, Not satisfied |
| One person coordinating pregnancy care | ‘*Was there one person who coordinated your pregnancy care and provided the majority of your pregnancy check-ups?*’ Women indicating ‘Yes, my GP’*,* ‘Yes, my midwife’, ‘Yes, my obstetrician’ or ‘Yes, other’ were coded *‘Yes’*. Coded *‘No’* if indicated ‘No’ or ‘I only had one pregnancy check-up’. | No, Yes |
| Given after hours contact details of a care provider during pregnancy | ‘*During your pregnancy, did you have the name and contact details of a someone you could get in touch with at any hour if worried?*’ Women could select all that apply, with response options of ‘I had the name and contact details of my care provider’, ‘I had the details of my hospital, clinic, or health service’, I had the details of a telephone support service or helpline’, ‘I had the details of someone else’ with an open-text area to specify, and ‘No’. Women who indicated ‘I had the name and contact details of my care provider’ were coded as *‘Given contact details of a named care provider’* regardless of what other responses they selected. Of the remaining women who had not indicated ‘I had the name and contact details of my care provider’, women who indicated ‘I had the details of my hospital, clinic, or health service’ were coded as *‘Given contact details of a service or facility’* regardless of what other responses they selected. All other responses that did not include ‘I had the name and contact details of my care provider’ or ‘I had the details of my hospital, clinic, or health service’ were coded as no. | Not given contact details of a care provider, Given contact details of a named care provider, Given contact details of a service or facility |
| **Maternal Experiences During Labour/Birth** | |  |
| A known care provider during labour/birth | Women were asked if they had met their care providers for labour/birth before they went into labour. Women indicating ‘*Yes, all of them*’ or ‘*Yes, some of them*’ were coded as ‘At least one known carer’ and those indicating ‘*No*’ were coded as ‘No known carers’. | At least one known carer, No known carers |
| Continuity of care throughout labour/birth | ‘*Was there at least one maternity care provider who cared for you right through your birthing experience (including labour)?*’ | No, Yes |
| Mobility during labour | ‘*During your labour were you able to move around and choose the position that made you most comfortable?*’ Women who did not indicate ‘Yes, all of the time’ were coded as *‘Not all of the time’*. | Yes, all of the time; Not all of the time |
| Support people made to feel welcome during labour | *‘Were all of your support people (e.g., partner, husband, companion) made to feel welcome during your labour?* Coded as *‘Yes’* if indicated ‘Yes’ and all other responses were coded as *‘No’.* Coded as *‘Yes’* if indicated ‘Yes’ and all other responses were coded as *‘No’.* | During labour:  No, Yes |
| Support people made to feel welcome during birth | *‘Were all of your support people (e.g., partner, husband, companion) made to feel welcome during your birth?* Coded as *‘Yes’* if indicated ‘Yes’ and all other responses were coded as *‘No’.* | No, Yes |
| Skin-to-skin contact first time holding baby | *‘The first time you held your baby. Did you have skin-to-skin contact (that is, was your baby straight on your skin and not wrapped, dressed or in a nappy)?’* | No, Yes |
| Perceived all medical procedures as necessary | *Do you feel that the medical procedures during your birth were necessary?* Coded as *‘All of them’* if indicated ‘All of them’ and coded as *‘Not all of them’* if indicated ‘Some of them’ or ‘None of them’. Women who did not receive medical procedures were excluded from analyses specific to this outcome. | All of them, Not all of them |
| **Maternal Experiences During Postpartum Care** | |  |
| Satisfied with the length of hospital stay | Women were asked if they felt that their length of hospital stay after birth was ‘Too long’, ‘Too short’, or ‘About the right amount of time’. Coded as *‘Yes’* if indicated ‘About the right amount of time’ and *‘No’* for all other responses. | Satisfied, Not satisfied |
| Support people made to feel welcome after birth | *‘Were all of your support people (e.g., partner, husband, companion) made to feel welcome after birth?* Coded as *‘Yes’* if indicated ‘Yes’ and all other responses were coded as *‘No’.* | No, Yes |
| Support people made to feel welcome overnight | *‘Were all of your support people (e.g., partner, husband, companion) made to feel welcome overnight?* Coded as *‘Yes’* if indicated ‘Yes’ and *‘No’* if indicated *‘No’.* Women who did not stay in the hospital or birth centre overnight were excluded from analyses specific to this outcome. | No, Yes |
| Given after hours contact details of a care provider at home after birth | ‘*When you were at home after the birth of your baby, did you have the name and contact details of a someone you could get in touch with at any hour if worried?*’ Women could select all that apply, with response options of ‘I had the name and contact details of my care provider’, ‘I had the details of my hospital, clinic, or health service’, I had the details of a telephone support service or helpline’, ‘I had the details of someone else’ with an open-text area to specify, and ‘No’. Women who indicated ‘I had the name and contact details of my care provider’ were coded as *‘Given contact details of a named care provider’* regardless of what other responses they selected. Of the remaining women who had not indicated ‘I had the name and contact details of my care provider’, women who indicated ‘I had the details of my hospital, clinic, or health service’ were coded as *‘Given contact details of a service or facility’* regardless of what other responses they selected. All other responses that did not include ‘I had the name and contact details of my care provider’ or ‘I had the details of my hospital, clinic, or health service’ were coded as no. | Not given contact details of a care provider, Given contact details of a named care provider, Given contact details of a service or facility |
| Contacted by a nurse or midwife within first 7 days of arriving home | Women were asked if they had been contacted at home by a nurse or midwife after having their baby. Coded as *‘Visited at home or telephoned’* if indicated ‘Yes, I was telephone by a midwife or nurse’ and/or ‘Yes, I was visited at home by a midwife or nurse’ and *‘Not contacted’* if indicated ‘No’ to ‘telephone by a midwife or nurse’ and ‘visited at home by a midwife or nurse’. Missing data was retained as a level of the variable due to greater than 5% missing. | Not contacted, Visited at home or telephoned, Missing data |
| Confident to care for baby after birth at home | *‘When you first had your new baby at home, how confident did you feel about looking after him or her?’* Coded as *‘Confident’* if indicated ‘Extremely confident’, ‘Fairly confident’ or ‘confident’. Coded as *‘Not confident’* if indicated ‘Not very confident’ or ‘Not at all confident’. Women who indicated that their baby had not yet come home were coded as missing. | Confident, Not confident |
| **Interpersonal Quality of Care** | |  |
| Women were separately asked about their care during pregnancy, labour/birth, postpartum care in hospital, and postpartum care after going home. Response options were ‘Not at all’, ‘Some of the time’, ‘Most of the time’ and ‘All of the time’. A separate dichotomous variable was created for each item for each period of care. Women who did not receive postpartum care at home were excluded from analyses of care during postpartum care at home. | |  |
| *‘When I saw care providers during my pregnancy, they…*  *‘When I saw care providers during my labour/birth, they…*  *‘When I saw care providers in hospital or birth centre after my birth, they…*  *‘When I saw care providers after going home (or since having my baby at home), they…* | |  |
| Care providers communicated well with other care providers | *…Communicated well with my other care providers’.* Coded *‘All of the time’* if indicated ‘All of the time’ and all other responses were coded as *‘Not all of the time’*. | All of the time, Not all of the time, |
| Care providers worked well as a team | *…Worked well as a team’.* Coded *‘All of the time’* if indicated ‘All of the time’ and all other responses were coded as *‘Not all of the time’*. | All of the time, Not all of the time |
| Care providers used language mothers could understand | *…Talked to me in a way I could understand’.* Coded *‘All of the time’* if indicated ‘All of the time’ and all other responses were coded as *‘Not all of the time’*. | All of the time, Not all of the time |
| Care providers treated mothers with respect | *…Treated me with respect’.* Coded *‘All of the time’* if indicated ‘All of the time’ and all other responses were coded as *‘Not all of the time’*. | All of the time, Not all of the time |
| Care providers talked to mothers with kindness and understanding | *…Treated me with kindness and understanding’.* Coded *‘All of the time’* if indicated ‘All of the time’ and all other responses were coded as *‘Not all of the time’*. | All of the time, Not all of the time |
| Care providers treated mothers as an individual | *…Treated me as an individual’.* Coded *‘All of the time’* if indicated ‘All of the time’ and all other responses were coded as *‘Not all of the time’*. | All of the time, Not all of the time |
| Care providers were open and honest | *…Were open and honest’.* Coded *‘All of the time’* if indicated ‘All of the time’ and all other responses were coded as *‘Not all of the time’*. | All of the time, Not all of the time |
| Care providers respected mothers’ privacy | *…Respected my privacy’.* Coded *‘All of the time’* if indicated ‘All of the time’ and all other responses were coded as *‘Not all of the time’*. | All of the time, Not all of the time |
| Care providers respected mothers’ decisions | *…Respected my decisions’.* Coded *‘All of the time’* if indicated ‘All of the time’ and all other responses were coded as *‘Not all of the time’*. | All of the time, Not all of the time |
| Care providers genuinely care about mothers’ wellbeing | *…Genuinely cared about my wellbeing’.* Coded *‘All of the time’* if indicated ‘All of the time’ and all other responses were coded as *‘Not all of the time’*. | All of the time, Not all of the time |
| *‘Thinking about your pregnancy, how often did you…*  *‘Thinking about your labour/birth, how often did you…*  *‘Thinking about your time in the hospital or birth centre after birth, how often did you…*  *‘When I saw care providers after going home (or since having my baby at home), they…* | |  |
| Mothers were confident in the skills of care providers | *…Feel confidence in the skills of your care providers?’* Coded *‘All of the time’* if indicated ‘All of the time’ and all other responses were coded as *‘Not all of the time’*. | All of the time, Not all of the time |
| Mothers knew what was happening | *… Know what was happening?’* Coded *‘All of the time’* if indicated ‘All of the time’ and all other responses were coded as *‘Not all of the time’*. | All of the time, Not all of the time |
| Mothers felt comfortable asking questions | *…Feel comfortable asking questions?’* Coded *‘All of the time’* if indicated ‘All of the time’ and all other responses were coded as *‘Not all of the time’*. | All of the time, Not all of the time |
| Mothers felt in control | *… Feel in control?’* Coded *‘All of the time’* if indicated ‘All of the time’ and all other responses were coded as *‘Not all of the time’*. | All of the time, Not all of the time |
| Mothers felt safe | *…Feel safe?’* Coded *‘All of the time’* if indicated ‘All of the time’ and all other responses were coded as *‘Not all of the time’*. | All of the time, Not all of the time |
| Mothers felt like care providers were on their side | *… Feel like your care providers were on your side?’* Coded *‘All of the time’* if indicated ‘All of the time’ and all other responses were coded as *‘Not all of the time’*. | All of the time, Not all of the time |
| Mothers never received conflicting information/advice from different care providers | *…Received conflicting information and advice from different care providers?* Coded *‘Never’* if indicated ‘Not at all’ and all other responses were coded as *‘At least some of the time’.* | Never, At least some of the time |
| Mothers never wanted to be more involved in decisions | *…Want to be more involved in decisions?* Coded *‘Never’* if indicated ‘Not at all’ and all other responses were coded as *‘At least some of the time’.* | Never, At least some of the time |
| Mothers never wished care providers had more time to talk | *…Wish your care providers had more time to talk to you?’* Coded *‘Never’* if indicated ‘Not at all’ and all other responses were coded as *‘At least some of the time’.* | Never, At least some of the time |
| **Overall Quality of Care** | |  |
| Women were separately asked about how well they were looked after by their care provider(s) during pregnancy, labour/birth, postpartum care in hospital, and postpartum care after going home. Response options were ‘Very badly’, ‘Badly’, ‘Neither well nor badly’ and ‘Well’ or ‘Very well’. | |  |
| Very well looked after by care provider(s) during pregnancy | *‘Overall, how well were you looked after by your care provider(s) during pregnancy?’* Women who indicated ‘Very well’ were coded as *‘Very Well’* and all other responses were coded as *‘Not very well’*. | Very well, Not very well |
| Very well looked after by care provider(s) during labour/birth | *‘Overall, how well were you looked after by your care provider(s) during labour/birth?’* Women who indicated ‘Very well’ were coded as *‘Very Well’* and all other responses were coded as *‘Not very well’*. | Very well, Not very well |
| Very well looked after by care provider(s) during postpartum care in hospital | *‘Overall, how well were you looked after by your care provider(s) in the hospital or birth centre after your birth?’* Women who indicated ‘Very well’ were coded as *‘Very Well’* and all other responses were coded as *‘Not very well’*. | Very well, Not very well, |
| Very well looked after by care provider(s) during postpartum care at home | *‘Overall, how well were you looked after by your care provider(s) after going home (or since having your baby at home)?’* Women who indicated ‘Very well’ were coded as *‘Very Well’* and all other responses were coded as *‘Not very well’*. Women who indicated not receiving postpartum care at home were excluded from analyses specific to this outcome. | Very well, Not very well |

**References**

1. Chen A, Xie C, Vuong AM, Wu T, Defranco EA. Optimal gestational weight gain: Prepregnancy BMI specific influences on adverse pregnancy and infant health outcomes. J Perinatol. 2017 Apr 1;37(4):369–74. doi: 10.1038/jp.2016.267

2. Korda RJ, Joshy G, Paige E, Butler JRG, Jorm LR, Liu B, et al. The relationship between body mass index and hospitalisation rates, days in hospital and costs: Findings from a large prospective linked data study. PLoS One. 2015 Mar 4;10(3):e0118599. doi: 10.1371/journal.pone.0118599

3. Whitlock G, Lewington S, Sherliker P, Clarke R, Kromhout D. Body-mass index and cause-specific mortality in 900 000 adults: collaborative analyses of 57 prospective studies Prospective Studies. Lancet. 2009;373(9669):1083–96. doi: 10.1016/S0140

4. Australian Bureau of Statistics. Australian Standard Geographical Classification (ASGC). Cat. No. 1216.0. Canberra: ABS; 2011.
