# Supplementary material for: A direct comparison of patient-reported outcomes and experiences in alternative models of maternity care in Queensland, Australia
Source: PLoS One. 2022 Jul 12;17(7):e0271105. doi: 10.1371/journal.pone.0271105 (PMC9275696; doi:10.1371/journal.pone.0271105)
Supplement: S2 Table — (DOCX) [file pone.0271105.s002.docx]

**S2 Table: Frequencies and crude odds ratios for obstetric interventions and maternal and infant clinical outcomes by model of care.**

|  | Standard Public  (*n* = 510) | GP Shared  (*n* = 609) | Public Midwifery Continuity  (*n* = 362) | Private Obstetric  (*n* = 1321) | GP Shared Care^1^ | | Public Midwifery Continuity Care^1^ | | Private Obstetric Care^1^ | |
| --- | --- | --- | --- | --- | --- | --- | --- | --- | --- | --- |
|  | *n* (%) | *n* (%) | *n* (%) | *n* (%) | OR [99% CI] | *p* | OR [99% CI] | *p* | OR [99% CI] | *p* |
| **Maternal Outcomes** |  |  |  |  |  |  |  |  |  |  |
| Mode of birth |  |  |  |  |  |  |  |  |  |  |
| Unassisted vaginal birth | 288 (56.5) | 361 (59.3) | 254 (70.2) | 537 (40.7) | 1.12 [0.82-1.54] | .343 | 1.81 [1.25-2.64] | <.001 | 0.53 [0.40-0.69] | <.001 |
| Assisted vaginal birth | 61 (12.0) | 75 (12.3) | 30 (8.3) | 200 (15.1) | 1.03 [0.64-1.66] | .857 | 0.67 [0.36-1.22] | .082 | 1.31 [0.88-1.97] | .082 |
| Scheduled caesarean birth | 69 (13.5) | 90 (14.8) | 15 (4.1) | 415 (31.4) | 1.11 [0.71-1.73] | .551 | 0.28 [0.13-0.59] | <.001 | 2.93 [2.03-4.23] | <.001 |
| Unscheduled caesarean birth | 92 (18.0) | 83 (13.6) | 63 (17.4) | 169 (12.8) | 0.72 [0.47-1.10] | .044 | 0.96 [0.60-1.52] | .809 | 0.67 [0.46-0.96] | .004 |
| Induction of labour | 145 (28.2) | 153 (25.1) | 86 (23.8) | 408 (30.9) | 0.85 [0.60-1.20] | .213 | 0.78 [0.52-1.18] | .124 | 1.13 [0.84-1.51] | .305 |
| Epidural/spinal block | 131 (25.7) | 132 (21.7) | 88 (24.3) | 377 (28.5) | 0.80 [0.56-1.15] | .115 | 0.93 [0.62-1.40] | .644 | 1.16 [0.85-1.57] | .222 |
| Continuous fetal monitoring during labour | 220 (43.1) | 268 (44.0) | 142 (39.2) | 542 (41.0) | 1.04 [0.76-1.42] | .770 | 0.85 [0.59-1.22] | .248 | 0.92 [0.70-1.20] | .412 |
| Perineal status |  |  |  |  |  |  |  |  |  |  |
| Perineum intact | 272 (53.3) | 312 (51.2) | 187 (51.7) | 758 (57.4) | 0.92 [0.67-1.25] | .483 | 0.94 [0.66-1.33] | .625 | 1.18 [0.90-1.54] | .118 |
| Perineal trauma with no sutures | 42 (8.2) | 40 (6.6) | 46 (12.7) | 38 (2.9) | 0.78 [0.43-1.42] | .287 | 1.62 [0.90-2.90] | .032 | 0.33 [0.18-0.60] | <.001 |
| Sutured perineal trauma | 196 (38.4) | 257 (42.2) | 129 (35.6) | 525 (39.7) | 1.17 [0.85-1.60] | .201 | 0.89 [0.61-1.28] | .400 | 1.06 [0.80-1.39] | .607 |
| Type of perineal trauma |  |  |  |  |  |  |  |  |  |  |
| Episiotomy | 56 (11.0) | 73 (12.0) | 24 (6.6) | 202 (15.3) | 1.10 [0.68-1.80] | .600 | 0.58 [0.30-1.11] | .030 | 1.46 [0.97-2.22] | .018 |
| Perineal tear | 204 (40.0) | 252 (41.4) | 159 (43.9) | 432 (32.7) | 1.06 [0.77-1.45] | .640 | 1.18 [0.82-1.68] | .247 | 0.73 [0.55-0.96] | .003 |
| Perineal tear following episiotomy | 22 (4.3) | 28 (4.6) | 8 (2.2) | 71 (5.4) | 1.07 [0.51-2.27] | .819 | 0.50 [0.17-1.47] | .099 | 1.26 [0.66-2.40] | .355 |
| Experienced breastfeeding problems | 288 (56.5) | 334 (54.8) | 154 (42.5) | 735 (55.6) | 0.94 [0.89-1.28] | .585 | 0.57 [0.40-0.82] | <.001 | 0.97 [0.74-1.27] | .748 |
| Experienced depression after birth | 167 (32.7) | 162 (26.6) | 79 (21.8) | 363 (27.5) | 0.74 [0.53-1.05] | .025 | 0.57 [0.38-0.86] | <.001 | 0.78 [0.58-1.04] | .026 |
| Experienced anxiety after birth | 257 (50.4) | 283 (46.5) | 153 (42.3) | 691 (52.3) | 0.86 [0.63-1.17] | .191 | 0.72 [0.51-1.03] | .018 | 1.08 [0.83-1.41] | .462 |
| Diagnosed depression after birth | 52 (10.2) | 42 (6.9) | 28 (7.7) | 83 (6.3) | 0.65 [0.37-1.14] | .049 | 0.74 [0.39-1.39] | .216 | 0.59 [0.37-0.95] | .004 |
| Diagnosed anxiety after birth | 48 (9.4) | 38 (6.2) | 21 (5.8) | 88 (6.7) | 0.64 [0.36-1.15] | .049 | 0.59 [0.30-1.19] | .054 | 0.69 [0.42-1.11] | .045 |
| Maternal hospital re-admission | 24 (4.7) | 30 (4.9) | 12 (3.3) | 60 (4.5) | 1.05 [0.51-2.16] | .864 | 0.69 [0.27-1.76] | .311 | 0.96 [0.51-1.82] | .881 |
| **Infant Outcomes** |  |  |  |  |  |  |  |  |  |  |
| Preterm birth (<37 weeks) | 48 (9.4) | 30 (4.9) | 11 (3.0) | 102 (7.7) | 0.50 [0.27-0.93] | .004 | 0.30 [0.13-0.73] | <.001 | 0.81 [0.50-1.29] | .238 |
| Low infant birth weight (<2500g) | 29 (5.7) | 30 (4.9) | 7 (1.9) | 53 (4.0) | 0.86 [0.43-1.71] | .571 | 0.33 [0.10-0.98] | .009 | 0.69 [0.38-1.28] | .122 |
| Neonate admission to NICU | 127 (24.9) | 106 (17.4) | 44 (12.2) | 179 (13.6) | 0.64 [0.43-0.93] | .002 | 0.42 [0.26-0.68] | <.001 | 0.47 [0.34-0.66] | <.001 |
| Neonate’s length of stay in NICU† |  |  |  |  |  |  |  |  |  |  |
| < 48 hours | 45 (35.4) | 51 (48.1) | 17 (38.6) | 70 (39.1) | 1.69 [0.85-3.38] | .051 | 1.15 [0.45-2.91] | .703 | 1.17 [0.63-2.18] | .513 |
| 48 hours to 7 days | 56 (44.1) | 32 (30.2) | 21 (47.7) | 52 (29.1) | 0.55 [0.27-1.12] | .030 | 1.15 [0.47-2.86] | .676 | 0.52 [0.28-0.97] | .007 |
| > 7 days | 26 (20.5) | 23 (21.7) | 6 (13.6) | 57 (31.8) | 1.08 [0.47-2.47] | .819 | 0.61 [0.17-2.17] | .320 | 1.82 [0.90-3.66] | .029 |
| Infant hospital re-admission | 43 (8.4) | 48 (7.9) | 35 (9.7) | 82 (6.2) | 0.93 [0.53-1.63] | .738 | 1.16 [0.63-2.15] | .528 | 0.72 [0.43-1.19] | .092 |
| Breastfeeding at 13 weeks | 333 (65.3) | 415 (68.1) | 297 (82.0) | 1032 (78.1) | 1.14 [0.82-1.58] | .313 | 2.43 [1.59-3.72] | <.001 | 1.90 [1.41-2.55] | <.001 |
| **Maternal Outcomes** | *M* (*SD*) | *M* (*SD*) | *M* (*SD*) | *M* (*SD*) | OR [99% CI] | *p* | OR [99% CI] | *p* | OR [99% CI] | *p* |
| Vaginal examinations during labour | 2.43 (2.41) | 2.32 (2.46) | 2.35 (2.14) | 1.85 (1.87) | 0.89 [0.64-1.25] | .381 | 0.92 [0.63-1.34] | .560 | 0.56 [0.41-0.75] | <.001 |
| Maternal length of hospital stay (nights) | 2.67 (1.64) | 2.52 (1.50) | 2.05 (1.73) | 4.21 (1.39) | 0.86 [0.68-1.08] | .084 | 0.54 [0.41-0.70] | <.001 | 4.67 [3.82-5.72] | .000 |

*Note*. NICU = neonatal intensive care unit.

^1^ vs. Standard Public Care

† Of the neonates admitted to the NICU (*n* = 456).
