# Supplementary material for: A direct comparison of patient-reported outcomes and experiences in alternative models of maternity care in Queensland, Australia
Source: PLoS One. 2022 Jul 12;17(7):e0271105. doi: 10.1371/journal.pone.0271105 (PMC9275696; doi:10.1371/journal.pone.0271105)
Supplement: S3 Table — (DOCX) [file pone.0271105.s003.docx]

**S3 Table. Frequencies and crude odds ratio for information provision and decision-making outcomes by model of care.**

|  | Standard Public  (*n* = 510) | GP Shared  (*n* = 609) | Public Midwifery Continuity  (*n* = 362) | Private Obstetric  (*n* = 1321) | GP Shared Care^1^ | | Public Midwifery Continuity Care^1^ | | Private Obstetric Care^1^ | |
| --- | --- | --- | --- | --- | --- | --- | --- | --- | --- | --- |
|  | *n* (%) | *n* (%) | *n* (%) | *n* (%) | OR [99% CI] | *p* | OR [99% CI] | *p* | OR [99% CI] | *p* |
| Pros and cons discussed for having/not having |  |  |  |  |  |  |  |  |  |  |
| Ultrasound scans | 273 (53.5) | 362 (59.4) | 206 (56.9) | 743 (56.2) | 1.27 [0.93-1.74] | .047 | 1.15 [0.80-1.64] | .324 | 1.12 [0.85-1.46] | .295 |
| Blood tests during pregnancy | 316 (62.0) | 408 (67.0) | 243 (67.1) | 878 (66.5) | 1.25 [0.90-1.72] | .079 | 1.25 [0.86-1.82] | .117 | 1.22 [0.92-1.61] | .070 |
| Caesarean birth | 325 (63.7) | 411 (67.5) | 231 (63.8) | 994 (75.2) | 1.18 [0.85-1.64] | .187 | 1.00 [0.70-1.45] | .979 | 1.73 [1.30-2.31] | <.001 |
| Induction of labour | 354 (69.4) | 416 (68.3) | 295 (81.5) | 881 (66.7) | 0.95 [0.68-1.33] | .692 | 1.94 [1.27-2.99] | <.001 | 0.88 [0.66-1.18] | .266 |
| Fetal monitoring during labour | 371 (72.7) | 449 (73.7) | 295 (81.5) | 858 (65.0) | 0.98 [0.68-1.43] | .898 | 0.58 [0.36-0.93] | .003 | 0.82 [0.59-1.14] | .120 |
| Vaginal examinations | 310 (60.8) | 395 (64.9) | 276 (76.2) | 668 (50.6) | 1.19 [0.87-1.64] | .160 | 2.07 [1.40-3.07] | <.001 | 0.66 [0.50-0.87] | <.001 |
| Epidural | 417 (81.8) | 500 (82.1) | 302 (83.4) | 1125 (85.2) | 1.02 [0.69-1.53] | .884 | 1.12 [0.70-1.80] | .525 | 1.28 [0.90-1.83] | .074 |
| Episiotomy | 242 (47.5) | 308 (50.6) | 219 (60.5) | 561 (42.5) | 1.13 [0.83-1.54] | .298 | 1.70 [1.18-2.43] | <.001 | 0.82 [0.62-1.07] | .054 |
| Syntocinon to birth placenta | 331 (64.9) | 422 (69.3) | 300 (82.9) | 651 (49.3) | 1.22 [0.88-1.70] | .119 | 2.62 [1.70-4.03] | <.001 | 0.53 [0.40-0.69] | <.001 |
| Procedures experienced without consent |  |  |  |  |  |  |  |  |  |  |
| Ultrasound scans | 36 (7.1) | 25 (4.1) | 27 (7.5) | 93 (7.0) | 0.56 [0.28-1.12] | .032 | 1.06 [0.54-2.10] | .822 | 1.00 [0.59-1.69] | .989 |
| Blood tests during pregnancy | 50 (9.8) | 48 (7.9) | 29 (8.0) | 113 (8.6) | 0.79 [0.46-1.36] | .258 | 0.80 [0.43-1.50] | .364 | 0.86 [0.54-1.36] | .400 |
| Caesarean birth | 75 (14.7) | 75 (12.3) | 31 (8.6) | 42 (3.2) | 0.82 [0.52-1.28] | .243 | 0.54 [0.30-0.97] | .007 | 0.19 [0.11-0.32] | <.001 |
| Induction of labour | 44 (8.6) | 42 (6.9) | 22 (6.1) | 71 (5.4) | 0.78 [0.44-1.39] | .269 | 0.66 [0.33-1.33] | .128 | 0.61 [0.37-1.03] | .015 |
| Missing data | 25 (4.9) | 27 (4.4) | 7 (1.9) | 91 (6.9) | 0.88 [0.42-1.84] | .659 | 0.37 [0.12-1.13] | .022 | 1.39 [0.76-2.52] | .161 |
| Fetal monitoring during labour | 113 (22.2) | 133 (21.8) | 51 (14.1) | 250 (18.9) | 1.05 [0.74-1.49] | .712 | 1.65 [1.07-2.54] | .003 | 0.69 [0.52-0.93] | .002 |
| Vaginal examinations | 48 (9.4) | 52 (8.5) | 16 (4.4) | 123 (9.3) | 0.90 [0.52-1.54] | .610 | 0.45 [0.21-9.96] | .006 | 0.99 [0.62-1.57] | .947 |
| Epidural | 38 (7.5) | 36 (5.9) | 14 (3.9) | 64 (4.8) | 0.78 [0.42-1.45] | .303 | 0.50 [0.22-1.14] | .030 | 0.63 [0.37-1.09] | .031 |
| Episiotomy | 84 (16.5) | 108 (17.7) | 43 (11.9) | 226 (17.1) | 1.09 [0.73-1.65] | .577 | 0.68 [0.41-1.15] | .059 | 1.05 [0.73-1.50] | .744 |
| Syntocinon to birth placenta | 44 (8.6) | 66 (10.8) | 38 (10.5) | 204 (15.4) | 1.29 [0.76-2.18] | .217 | 1.24 [0.68-2.26] | .352 | 1.93 [1.23-3.04] | <.001 |

^1^ vs. Standard Public Care
