# Supplementary material for: A direct comparison of patient-reported outcomes and experiences in alternative models of maternity care in Queensland, Australia
Source: PLoS One. 2022 Jul 12;17(7):e0271105. doi: 10.1371/journal.pone.0271105 (PMC9275696; doi:10.1371/journal.pone.0271105)
Supplement: S4 Table — (DOCX) [file pone.0271105.s004.docx]

**S4 Table. Frequencies and crude odd ratios for maternal experiences during pregnancy, labour/birth, and postpartum care by model of care.**

|  | Standard Public  (*n* = 510) | GP Shared  (*n* = 609) | Public Midwifery Continuity  (*n* = 362) | Private Obstetric  (*n* = 1321) | GP Shared Care^1^ | | Public Midwifery Continuity Care^1^ | | Private Obstetric Care^1^ | |
| --- | --- | --- | --- | --- | --- | --- | --- | --- | --- | --- |
|  | *n* (%) | *n* (%) | *n* (%) | *n* (%) | OR [99% CI] | *p* | OR [99% CI] | *p* | OR [99% CI] | *p* |
| **Pregnancy** |  |  |  |  |  |  |  |  |  |  |
| Able to choose gender of care provider | 33 (6.5) | 41 (6.7) | 48 (13.3) | 391 (29.6) | 1.04 [0.56-1.95] | .861 | 2.21 [1.20-4.07] | <.001 | 6.08 [3.73-9.91] | <.001 |
| Able to choose mode of birth | 254 (49.8) | 304 (49.9) | 143 (39.5) | 991 (75.0) | 1.01 [0.74-1.37] | .970 | 0.66 [0.46-0.94] | .003 | 3.03 [2.29-4.01] | <.001 |
| Satisfied with timing of booking appointment† | 387 (77.6) | 466 (79.0) | 274 (79.9) | 922 (88.9) | 1.09 [0.74-1.59] | .569 | 1.15 [0.74-1.79] | .419 | 2.32 [1.59-3.38] | <.001 |
| Satisfied with number of pregnancy check-ups | 417 (81.8) | 526 (86.4) | 319 (88.1) | 1198 (90.7) | 1.41 [0.93-2.16] | .036 | 1.66 [0.99-2.76] | .011 | 2.17 [1.48-3.19] | <.001 |
| One person coordinating pregnancy care | 297 (58.2) | 531 (87.2) | 319 (88.1) | 1294 (98.0) | 4.88 [3.31-7.20] | <.001 | 5.32 [3.30-8.58] | <.001 | 34.37 [19.80-59.67] | <.001 |
| Given after hours contact details of a care provider |  |  |  |  |  |  |  |  |  |  |
| A named care provider | 132 (25.9) | 229 (37.6) | 244 (67.4) | 981 (74.3) | 1.25 [0.64-2.44] | .389 | 7.16 [2.49-20.65] | <.001 | 6.06 [3.11-11.82] | <.001 |
| A hospital, clinic, or health service | 347 (68.0) | 337 (55.3) | 110 (30.4) | 302 (22.9) | 0.70 [0.37-1.33] | .150 | 1.23 [0.43-3.54] | .617 | 0.71 [0.37-1.37] | .178 |
| **Labour/Birth** |  |  |  |  |  |  |  |  |  |  |
| A known care provider during labour/birth | 270 (52.9) | 240 (39.4) | 291 (80.4) | 1183 (89.6) | 0.58 [0.42-0.79] | <.001 | 3.64 [2.42-5.49] | <.001 | 7.62 [5.50-10.55] | <.001 |
| Continuity of care throughout labour/birth | 291 (57.1) | 353 (58.0) | 275 (76.0) | 1128 (85.4) | 1.04 [0.76-1.42] | .760 | 2.38 [1.61-3.52] | <.001 | 4.40 [3.24-5.97] | <.001 |
| Mobility during labour | 177 (34.7) | 239 (39.2) | 200 (55.2) | 354 (26.8) | 1.22 [0.88-1.68] | .118 | 2.32 [1.62-3.34] | <.001 | 0.69 [0.52-0.92] | <.001 |
| Support people made to feel were welcome |  |  |  |  |  |  |  |  |  |  |
| During labour | 444 (87.1) | 540 (88.7) | 343 (94.8) | 1062 (80.4) | 1.16 [0.73-1.87] | .410 | 2.68 [1.34-5.38] | <.001 | 0.61 [0.42-0.90] | <.001 |
| During birth | 493 (96.7) | 583 (95.7) | 352 (97.2) | 1301 (98.5) | 0.77 [0.34-1.75] | .418 | 1.21 [0.43-3.44] | .632 | 2.24 [0.95-5.30] | .016 |
| Skin-to-skin contact first time holding baby | 395 (77.5) | 482 (79.1) | 318 (87.8) | 923 (69.9) | 1.11 [0.76-1.61] | .493 | 2.10 [1.28-3.46] | <.001 | 0.68 [0.49-0.92] | .001 |
| Perceived all medical procedures necessary‡ | 323 (78.8) | 409 (85.0) | 191 (78.6) | 1050 (90.2) | 1.53 [0.97-2.41] | .016 | 0.99 [0.60-1.65] | .957 | 2.48 [1.66-3.71] | <.001 |
| **Postpartum Care** |  |  |  |  |  |  |  |  |  |  |
| Support people made to feel welcome |  |  |  |  |  |  |  |  |  |  |
| After birth | 481 (94.3) | 573 (94.1) | 354 (97.8) | 1301 (98.5) | 0.96 [0.50-1.86] | .873 | 2.67 [0.94-7.58] | .016 | 3.92 [1.83-8.40] | <.001 |
| Overnight ¶ | 120 (30.2) | 151 (32.6) | 110 (45.5) | 1098 (91.2) | 1.12 [0.76-1.63] | .453 | 1.92 [1.24-2.97] | <.001 | 23.91 [16.28-35.12] | <.001 |
| Satisfied with the length of hospital stay | 364 (71.4) | 461 (75.7) | 276 (76.2) | 1131 (85.6) | 1.25 [0.88-1.77] | .102 | 1.29 [0.86-1.93] | .109 | 2.39 [1.73-3.30] | <.001 |
| Given after hours contact details of a care provider |  |  |  |  |  |  |  |  |  |  |
| A named care provider | 152 (29.8) | 255 (41.9) | 246 (68.0) | 784 (59.3) | 2.62 [1.29-5.33] | <.001 | 5.74 [2.29-14.38] | <.001 | 3.19 [1.80-5.67] | <.001 |
| A hospital, clinic, or health service | 319 (62.5) | 329 (54.0) | 105 (29.0) | 474 (35.9) | 1.61 [0.81-3.21] | .076 | 1.17 [0.46-2.95] | .667 | 0.92 [0.53-1.61] | .699 |
| Visited at home or telephoned after arriving home | 458 (89.8) | 545 (89.5) | 342 (94.5) | 291 (22.0) | 1.13 [0.62-2.07] | .602 | 4.73 [1.50-14.88] | <.001 | 0.02 [0.02-0.05] | <.001 |
| Missing data | 14 (2.7) | 24 (3.9) | 14 (3.9) | 36 (2.7) | 1.63 [0.57-4.63] | .229 | 6.33 [1.42-28.18] | .001 | 0.10 [0.05-0.25] | <.001 |
| Confident to care for baby after birth at home | 445 (87.3) | 549 (90.1) | 319 (88.1) | 1135 (85.9) | 1.34 [0.82-2.18] | .127 | 1.08 [0.63-1.86] | .702 | 0.89 [0.60-1.33] | .457 |
| **Pregnancy** | *M* (*SD*) | *M* (*SD*) | *M* (*SD*) | *M* (*SD*) | OR [99% CI] | *p* | OR [99% CI] | *p* | OR [99% CI] | *p* |
| Weeks gestation at first pregnancy check-up§ | 9.07 (4.60) | 8.08 (3.74) | 9.05 (4.62) | 7.74 (3.18) | 0.37 [0.21-0.67] | <.001 | 0.98 [0.50-1.92] | .933 | 0.26 [0.16-0.44] | <.001 |
| Weeks gestation at booking appointment† | 16.26 (5.64) | 17.74 (6.30) | 15.78 (4.84) | 17.48 (9.39) | 4.41 [1.37-14.27] | .001 | 0.62 [0.16-2.41] | .366 | 3.38 [1.18-9.66] | .003 |
| Number of pregnancy check-ups | 10.94 (5.28) | 11.66 (5.50) | 9.99 (4.22) | 11.95 (3.86) | 2.05 [1.01-4.15] | .009 | 0.39 [0.72-0.87] | .002 | 2.75 [1.49-5.09] | <.001 |

^1^ vs. Standard Public Care

† Of the women who had a booking appointment (*n* = 2469)

‡ Of the women who had medical procedures (*n* = 2298)

§ Of the women who had pregnancy check-ups (*n* = 2796)

¶ Of the women who stayed overnight (*n* = 2306)
