# Supplementary material for: A direct comparison of patient-reported outcomes and experiences in alternative models of maternity care in Queensland, Australia
Source: PLoS One. 2022 Jul 12;17(7):e0271105. doi: 10.1371/journal.pone.0271105 (PMC9275696; doi:10.1371/journal.pone.0271105)
Supplement: S5 Table — (DOCX) [file pone.0271105.s005.docx]

**S5 Table. Frequencies and crude odds ratios of maternal experiences of interpersonal and overall quality of care by maternity model of care.**

|  | Standard Public  (*n* = 510) | GP Shared  (*n* = 609) | Public Midwifery Continuity  (*n* = 362) | Private Obstetric  (*n* = 1321) | GP Shared Care^1^ | | Public Midwifery Continuity Care^1^ | | Private Obstetric Care^1^ | |
| --- | --- | --- | --- | --- | --- | --- | --- | --- | --- | --- |
|  | *n* (%) | *n* (%) | *n* (%) | *n* (%) | OR [99% CI] | *p* | OR [99% CI] | *p* | OR [99% CI] | *p* |
| Care providers communicated well with other care providers all of the time |  |  |  |  |  |  |  |  |  |  |
| During pregnancy | 173 (33.9) | 193 (31.7) | 188 (51.9) | 884 (66.9) | 0.90 [0.65-1.26] | .428 | 2.11 [1.47-3.03] | <.001 | 3.94 [2.97-5.24] | <.001 |
| During labour/birth | 295 (57.8) | 347 (57.0) | 267 (73.8) | 1022 (77.4) | 0.97 [0.71-1.32] | .771 | 2.05 [1.39-3.01] | <.001 | 2.49 [1.87-3.32] | <.001 |
| During postpartum care in hospital | 195 (38.2) | 225 (36.9) | 209 (57.7) | 684 (51.8) | 0.95 [0.69-1.30] | .657 | 2.21 [1.54-3.17] | <.001 | 1.74 [1.32-2.28] | <.001 |
| During postpartum care after going home† | 228 (46.7) | 245 (41.2) | 222 (62.7) | 572 (47.6) | 0.80 [0.58-1.10] | .071 | 1.92 [1.33-2.77] | <.001 | 1.04 [0.79-1.37] | .735 |
| Care providers worked well as a team all of the time |  |  |  |  |  |  |  |  |  |  |
| During pregnancy | 202 (39.6) | 245 (40.2) | 243 (67.1) | 955 (72.3) | 1.03 [0.75-1.41] | .832 | 3.11 [2.15-4.51] | <.001 | 3.98 [3.00-5.27] | <.001 |
| During labour/birth | 331 (64.9) | 409 (67.2) | 281 (77.6) | 1076 (81.5) | 1.11 [0.80-1.53] | .427 | 1.88 [1.25-2.81] | <.001 | 2.38 [1.76-3.21] | <.001 |
| During postpartum care in hospital | 211 (41.4) | 252 (41.4) | 215 (59.4) | 715 (54.1) | 1.00 [0.73-1.37] | .998 | 2.07 [1.45-2.97] | <.001 | 1.67 [1.27-2.19] | <.001 |
| During postpartum care after going home† | 247 (50.6) | 276 (46.5) | 243 (68.6) | 624 (52.0) | 0.85 [0.62-1.16] | .174 | 2.14 [1.47-3.11] | <.001 | 1.06 [0.80-1.39] | .617 |
| Care providers used language women could understand all of the time |  |  |  |  |  |  |  |  |  |  |
| During pregnancy | 325 (63.7) | 398 (65.4) | 305 (84.3) | 1116 (84.5) | 1.07 [0.78-1.48] | .571 | 3.05 [1.96-4.73] | <.001 | 3.10 [2.28-4.22] | <.001 |
| During labour/birth | 367 (72.0) | 446 (73.2) | 308 (85.1) | 1132 (85.7) | 1.07 [0.75-1.51] | .634 | 2.22 [1.41-3.51] | <.001 | 2.33 [1.69-3.23] | <.001 |
| During postpartum care in hospital | 293 (57.5) | 357 (58.6) | 265 (73.2) | 973 (73.7) | 1.05 [0.77-1.44] | .693 | 2.02 [1.38-2.97] | <.001 | 2.07 [1.56-2.74] | <.001 |
| During postpartum care after going home† | 339 (69.5) | 422 (71.0) | 294 (83.1) | 882 (73.4) | 1.08 [0.76-1.52] | .572 | 2.15 [1.38-3.36] | <.001 | 1.22 [0.90-1.65] | .099 |
| Care providers treated women with respect all of the time |  |  |  |  |  |  |  |  |  |  |
| During pregnancy | 356 (69.8) | 455 (74.7) | 316 (87.3) | 1195 (90.5) | 1.28 [0.91-1.81] | .067 | 2.97 [1.85-4.79] | <.001 | 4.10 [2.90-5.80] | <.001 |
| During labour/birth | 378 (74.1) | 462 (76.0) | 319 (88.1) | 1180 (89.3) | 1.11 [0.78-1.58] | .462 | 2.59 [1.58-4.24] | <.001 | 2.92 [2.07-4.14] | <.001 |
| During postpartum care in hospital | 300 (58.8) | 379 (62.2) | 269 (74.3) | 989 (74.9) | 1.15 [0.84-1.58] | .245 | 2.03 [1.38-2.98] | <.001 | 2.09 [1.57-2.77] | <.001 |
| During postpartum care after going home† | 351 (71.9) | 440 (74.1) | 303 (85.6) | 912 (75.9) | 1.12 [0.78-1.59] | .428 | 2.32 [1.45-3.70] | <.001 | 1.23 [0.90-1.68] | .086 |
| Care providers talked to women with kindness and understanding all of the time |  |  |  |  |  |  |  |  |  |  |
| During pregnancy | 335 (65.7) | 450 (73.9) | 310 (85.6) | 1151 (87.1) | 1.48 [1.06-2.07] | .003 | 3.11 [1.98-4.91] | <.001 | 3.54 [2.57-4.87] | <.001 |
| During labour/birth | 374 (73.3) | 456 (74.9) | 314 (86.7) | 1155 (87.4) | 1.08 [0.76-1.54] | .557 | 2.38 [1.48-3.83] | <.001 | 2.53 [1.81-3.54] | <.001 |
| During postpartum care in hospital | 292 (57.3) | 370 (60.8) | 263 (72.7) | 947 (71.7) | 1.16 [0.84-1.58] | .236 | 1.98 [1.36-2.90] | <.001 | 1.89 [1.43-2.50] | <.001 |
| During postpartum care after going home† | 353 (72.3) | 440 (74.1) | 303 (85.6) | 899 (74.9) | 1.09 [0.77-1.56] | .520 | 2.27 [1.42-3.63] | <.001 | 1.14 [0.83-1.56] | .284 |
| Care providers treated women as an individual all of the time |  |  |  |  |  |  |  |  |  |  |
| During pregnancy | 332 (65.1) | 415 (68.1) | 303 (83.7) | 1122 (84.9) | 1.15 [0.83-1.59] | .281 | 2.75 [1.78-4.27] | <.001 | 3.02 [2.22-4.12] | <.001 |
| During labour/birth | 382 (74.9) | 456 (74.9) | 313 (86.5) | 1138 (86.1) | 1.00 [0.70-1.43] | .992 | 2.14 [1.33-3.44] | <.001 | 2.08 [1.49-2.91] | <.001 |
| During postpartum care in hospital | 293 (57.5) | 364 (59.8) | 265 (73.2) | 949 (71.8) | 1.10 [0.80-1.51] | .433 | 2.02 [1.38-2.97] | <.001 | 1.89 [1.43-2.50] | <.001 |
| During postpartum care after going home† | 347 (71.1) | 437 (73.6) | 300 (84.7) | 892 (74.3) | 1.13 [0.80-1.61] | .367 | 2.26 [1.43-3.57] | <.001 | 1.17 [0.86-1.60] | .183 |
| Care providers were open and honest all of the time |  |  |  |  |  |  |  |  |  |  |
| During pregnancy | 349 (68.4) | 444 (72.9) | 317 (87.6) | 1155 (87.4) | 1.24 [0.88-1.74] | .101 | 3.25 [2.02-5.24] | <.001 | 3.21 [2.32-4.45] | <.001 |
| During labour/birth | 373 (73.1) | 462 (75.9) | 318 (87.8) | 1141 (86.4) | 1.15 [0.81-1.65] | .297 | 2.66 [1.63-4.32] | <.001 | 2.33 [1.67-3.24] | <.001 |
| During postpartum care in hospital | 310 (60.7) | 385 (63.2) | 273 (75.4) | 993 (75.2) | 1.11 [0.81-1.53] | .403 | 1.98 [1.34-2.93] | <.001 | 1.95 [1.47-2.60] | <.001 |
| During postpartum care after going home† | 356 (73.0) | 440 (74.1) | 307 (86.7) | 924 (76.9) | 1.06 [0.74-1.51] | .677 | 2.42 [1.50-3.92] | <.001 | 1.24 [0.90-1.70] | .083 |
| Care providers respected women’s privacy all of the time |  |  |  |  |  |  |  |  |  |  |
| During pregnancy | 389 (76.3) | 489 (80.3) | 327 (90.3) | 1214 (91.9) | 1.27 [0.87-1.85] | .104 | 2.91 [1.71-4.94] | <.001 | 3.53 [2.43-5.13] | <.001 |
| During labour/birth | 386 (75.7) | 472 (77.5) | 315 (87.0) | 1166 (88.3) | 1.11 [0.78-1.59] | .474 | 2.15 [1.33-3.49] | <.001 | 2.42 [1.71-3.41] | <.001 |
| During postpartum care in hospital | 303 (59.4) | 374 (61.4) | 274 (75.7) | 1002 (75.9) | 1.09 [0.79-1.49] | .495 | 2.13 [1.44-3.15] | <.001 | 2.15 [1.61-2.85] | <.001 |
| During postpartum care after going home† | 366 (75.0) | 447 (75.3) | 309 (87.3) | 943 (78.5) | 1.01 [0.70-1.46] | .924 | 2.29 [1.40-3.74] | <.001 | 1.22 [0.88-1.69] | .117 |
| Care providers respected women’s decisions all of the time |  |  |  |  |  |  |  |  |  |  |
| During pregnancy | 334 (65.5) | 449 (73.7) | 307 (84.8) | 1132 (85.7) | 1.48 [1.06-2.07] | .003 | 2.94 [1.88-4.60] | <.001 | 3.16 [2.31-4.32] | <.001 |
| During labour/birth | 371 (72.7) | 447 (73.4) | 300 (82.9) | 1120 (84.8) | 1.03 [0.73-1.47] | .806 | 1.81 [1.17-2.82] | <.001 | 2.09 [1.51-2.89] | <.001 |
| During postpartum care in hospital | 293 (57.5) | 352 (57.8) | 259 (71.5) | 947 (71.7) | 1.01 [0.74-1.39] | .906 | 1.86 [1.28-2.72] | <.001 | 1.88 [1.42-2.48] | <.001 |
| During postpartum care after going home† | 344 (70.5) | 427 (71.9) | 294 (83.1) | 884 (73.6) | 1.07 [0.76-1.52] | .614 | 2.05 [1.31-3.20] | <.001 | 1.17 [0.86-1.59] | .193 |
| Care providers genuinely cared about women’s wellbeing all of the time |  |  |  |  |  |  |  |  |  |  |
| During pregnancy | 341 (66.9) | 435 (71.4) | 313 (86.5) | 1160 (87.8) | 1.24 [0.89-1.73] | .099 | 3.17 [1.99-5.04] | <.001 | 3.57 [2.58-4.94] | <.001 |
| During labour/birth | 387 (75.9) | 464 (76.2) | 312 (86.2) | 1180 (89.3) | 1.02 [0.71-1.46] | .904 | 1.98 [1.23-3.19] | <.001 | 2.66 [1.87-3.78] | <.001 |
| During postpartum care in hospital | 299 (58.6) | 370 (60.8) | 264 (72.9) | 966 (73.1) | 1.09 [0.80-1.50] | .470 | 1.90 [1.30-2.79] | <.001 | 1.92 [1.45-2.54] | <.001 |
| During postpartum care after going home† | 357 (73.2) | 434 (73.1) | 300 (84.7) | 885 (73.7) | 1.00 [0.70-1.42] | .973 | 2.04 [1.28-3.24] | <.001 | 1.03 [0.75-1.41] | .822 |
| Women were confident in the skills of care providers all of the time |  |  |  |  |  |  |  |  |  |  |
| During pregnancy | 250 (49.0) | 305 (50.1) | 255 (70.4) | 1109 (84.0) | 1.04 [0.77-1.42] | .723 | 2.48 [1.71-3.60] | <.001 | 5.44 [4.04-7.34] | <.001 |
| During labour/birth | 328 (64.3) | 398 (65.4) | 278 (76.8) | 1101 (83.3) | 1.05 [0.76-1.45] | .717 | 1.84 [1.23-2.74] | <.001 | 2.78 [2.05-3.77] | <.001 |
| During postpartum care in hospital | 263 (51.6) | 293 (48.1) | 227 (62.7) | 775 (58.7) | 0.87 [0.64-1.19] | .249 | 1.58 [1.10-2.27] | .001 | 1.33 [1.02-1.75] | .006 |
| During postpartum care after going home† | 266 (54.5) | 336 (56.6) | 251 (70.9) | 715 (59.5) | 1.09 [0.79-1.49] | .498 | 2.03 [1.39-2.98] | <.001 | 1.23 [0.93-1.62] | .058 |
| Women knew what was happening all of the time |  |  |  |  |  |  |  |  |  |  |
| During pregnancy | 208 (40.8) | 289 (47.5) | 226 (62.4) | 925 (70.0) | 1.31 [0.96-1.79] | .025 | 2.41 [1.68-3.47] | <.001 | 3.39 [2.57-4.48] | <.001 |
| During labour/birth | 226 (44.3) | 287 (47.1) | 204 (56.4) | 778 (58.9) | 1.12 [0.82-1.53] | .347 | 1.62 [1.14-2.32] | <.001 | 1.80 [1.37-2.36] | <.001 |
| During postpartum care in hospital | 201 (39.4) | 221 (36.3) | 187 (51.7) | 670 (50.7) | 0.88 [0.64-1.20] | .283 | 1.64 [1.15-2.35] | <.001 | 1.58 [1.20-2.08] | <.001 |
| During postpartum care after going home† | 290 (59.4) | 337 (56.7) | 241 (68.1) | 706 (58.8) | 0.90 [0.65-1.23] | .372 | 1.46 [1.00-2.12 | .010 | 0.97 [0.74-1.29] | .808 |
| Women felt comfortable asking questions all of the time |  |  |  |  |  |  |  |  |  |  |
| During pregnancy | 301 (59.0) | 382 (62.7) | 290 (80.1) | 1065 (80.6) | 1.17 [0.85-1.60] | .206 | 2.80 [1.85-4.22] | <.001 | 2.89 [2.16-3.87] | <.001 |
| During labour/birth | 316 (62.0) | 406 (66.7) | 272 (75.1) | 1031 (78.0) | 1.23 [0.89-1.70] | .101 | 1.86 [1.25-2.75] | <.001 | 2.18 [1.63-2.92] | <.001 |
| During postpartum care in hospital | 256 (50.2) | 303 (49.8) | 232 (64.1) | 817 (61.8) | 0.98 [0.72-1.34] | .883 | 1.77 [1.23-2.55] | <.001 | 1.61 [1.23-2.11] | <.001 |
| During postpartum care after going home† | 325 (66.6) | 389 (65.5) | 279 (78.8) | 821 (68.4) | 0.95 [0.68-1.33] | .701 | 1.87 [1.23 [2.83] | <.001 | 1.08 [0.81-1.45] | .482 |
| Women felt in control all of the time |  |  |  |  |  |  |  |  |  |  |
| During pregnancy | 191 (37.5) | 272 (44.7) | 224 (61.9) | 791 (59.9) | 1.35 [0.98-1.85] | .015 | 2.71 [1.88-3.91] | <.001 | 2.49 [1.89-3.29] | <.001 |
| During labour/birth | 166 (32.5) | 240 (39.4) | 178 (49.2) | 607 (46.0) | 1.35 [0.98-1.86] | .018 | 2.01 [1.39-2.89] | <.001 | 1.76 [1.33-2.34] | <.001 |
| During postpartum care in hospital | 179 (35.1) | 219 (36.0) | 192 (53.0) | 632 (47.8) | 1.04 [0.75-1.43] | .764 | 2.09 [1.46-3.00] | <.001 | 1.70 [1.29-2.24] | <.001 |
| During postpartum care after going home† | 272 (55.7) | 332 (55.9) | 237 (66.9) | 658 (54.8) | 1.01 [0.73-1.38] | .959 | 1.61 [1.11-2.34] | .001 | 0.96 [0.73-1.27] | .722 |
| Women never received conflicting information/advice from different care providers |  |  |  |  |  |  |  |  |  |  |
| During pregnancy | 190 (37.3) | 245 (40.2) | 184 (50.8) | 867 (65.6) | 1.13 [0.83-1.56] | .309 | 1.74 [1.22-2.49] | <.001 | 3.22 [2.43-4.25] | <.001 |
| During labour/birth | 308 (60.4) | 397 (65.2) | 260 (71.8) | 1016 (76.9) | 1.23 [0.89-1.69] | .098 | 1.67 [1.14-2.45] | <.001 | 2.19 [1.64-2.91] | <.001 |
| During postpartum care in hospital | 199 (39.0) | 251 (41.2) | 171 (47.2) | 508 (38.5) | 1.10 [0.80-1.50] | .456 | 1.40 [0.98-2.00] | .016 | 0.98 [0.74-1.29] | .824 |
| During postpartum care after going home† | 217 (44.5) | 276 (46.5) | 197 (55.6) | 561 (46.7) | 1.08 [0.79-1.49] | .512 | 1.57 [1.09-2.25] | .001 | 1.10 [0.83-1.45] | .402 |
| Women felt safe all of the time |  |  |  |  |  |  |  |  |  |  |
| During pregnancy | 328 (64.3) | 395 (64.9) | 293 (80.9) | 1085 (82.1) | 1.02 [0.74-1.42] | .849 | 2.36 [1.55-3.58] | <.001 | 2.55 [1.89-3.45] | <.001 |
| During labour/birth | 335 (65.7) | 407 (66.8) | 283 (78.2) | 1043 (79.0) | 1.05 [0.76-1.46] | .687 | 1.87 [1.25-2.81] | <.001 | 1.96 [1.46-2.64] | <.001 |
| During postpartum care in hospital | 327 (64.1) | 378 (62.1) | 277 (76.5) | 1029 (77.9) | 0.92 [0.67-1.26] | .480 | 1.82 [1.23-2.72] | <.001 | 1.97 [1.47-2.64] | <.001 |
| During postpartum care after going home† | 341 (69.9) | 417 (70.2) | 289 (81.6) | 871 (72.5) | 1.02 [0.72-1.43] | .908 | 1.92 [1.24-2.96] | <.001 | 1.14 [0.84-1.54] | .274 |
| Women never wanted to be more involved in decisions |  |  |  |  |  |  |  |  |  |  |
| During pregnancy | 214 (42.0) | 281 (46.1) | 209 (57.7) | 861 (65.2) | 1.19 [0.87-1.62] | .161 | 1.89 [1.32-2.70] | <.001 | 2.59 [1.97-3.41] | <.001 |
| During labour/birth | 266 (52.2) | 321 (52.7) | 208 (57.5) | 891 (67.4) | 1.02 [0.75-1.39] | .854 | 1.24 [0.87-1.77] | .122 | 1.90 [1.45-2.50] | <.001 |
| During postpartum care in hospital | 218 (42.7) | 289 (47.5) | 196 (54.1) | 829 (62.8) | 1.21 [0.89-1.65] | .115 | 1.58 [1.11-2.26] | <.001 | 2.26 [1.72-2.97] | <.001 |
| During postpartum care after going home† | 271 (55.5) | 347 (58.4) | 223 (63.0) | 803 (66.9) | 1.13 [0.82-1.55] | .340 | 1.36 [0.94-1.97] | .030 | 1.62 [1.22-2.14] | <.001 |
| Women felt like care providers were on their side all of the time |  |  |  |  |  |  |  |  |  |  |
| During pregnancy | 279 (54.7) | 379 (62.2) | 284 (78.5) | 1087 (82.3) | 1.36 [1.00-1.87] | .011 | 3.02 [2.02-4.50] | <.001 | 3.85 [2.86-5.17] | <.001 |
| During labour/birth | 337 (66.1) | 435 (71.4) | 279 (77.1) | 1106 (83.7) | 1.28 [0.92-1.79] | .054 | 1.73 [1.15-2.58] | <.001 | 2.64 [1.94-3.59] | <.001 |
| During postpartum care in hospital | 259 (50.8) | 307 (50.4) | 236 (65.2) | 827 (62.6) | 0.99 [0.72-1.34] | .901 | 1.82 [1.26-2.61] | <.001 | 1.62 [1.24-2.13] | <.001 |
| During postpartum care after going home† | 309 (63.3) | 391 (65.8) | 270 (76.3) | 814 (67.8) | 1.12 [0.80-1.55] | .391 | 1.86 [1.25-2.79] | <.001 | 1.22 [0.91[1.63] | .079 |
| Women never wished care providers had more time to talk |  |  |  |  |  |  |  |  |  |  |
| During pregnancy | 182 (35.7) | 238 (39.1) | 205 (56.6) | 772 (58.4) | 1.16 [0.84-1.59] | .243 | 2.35 [1.64-3.38] | <.001 | 2.53 [1.92-3.35] | <.001 |
| During labour/birth | 246 (48.2) | 337 (55.3) | 237 (65.5) | 857 (64.9) | 1.33 [0.98-1.81] | .018 | 2.04 [1.41-2.93] | <.001 | 1.98 [1.51-2.60] | <.001 |
| During postpartum care in hospital | 169 (33.1) | 206 (33.8) | 177 (48.9) | 627 (47.5) | 1.03 [0.74-1.43] | .808 | 1.93 [1.34-2.78] | <.001 | 1.82 [1.38-2.41] | <.001 |
| During postpartum care after going home† | 216 (44.3) | 264 (44.4) | 196 (55.4) | 623 (51.9) | 1.01 [0.73-1.38] | .952 | 1.56 [1.09-2.24] | .001 | 1.36 [1.03-1.79] | .005 |
| Women were looked after very well by care providers |  |  |  |  |  |  |  |  |  |  |
| During pregnancy | 269 (52.7) | 333 (54.7) | 281 (77.6) | 1092 (82.7) | 1.08 [0.79-1.47] | .518 | 3.11 [2.09-4.62] | <.001 | 4.27 [3.18-5.74] | <.001 |
| During labour and birth | 319 (62.5) | 418 (68.6) | 282 (77.9) | 1112 (84.2) | 1.31 [0.95-1.82] | .033 | 2.11 [1.41-3.16] | <.001 | 3.19 [2.35-4.32] | <.001 |
| During postpartum care in hospital | 227 (44.5) | 277 (45.5) | 215 (59.4) | 844 (63.9) | 1.04 [0.76-1.42] | .744 | 1.82 [1.27-2.61] | <.001 | 2.21 [1.68-2.90] | <.001 |
| During postpartum care after going home† | 250 (51.2) | 288 (48.5) | 234 (66.1) | 521 (43.4) | 0.90 [0.65-1.23] | .369 | 1.86 [1.28-2.69] | <.001 | 0.73 [0.55-0.96] | .003 |

^1^ vs. Standard Public Care

† Of the women who received postpartum care at home (*n* = 2637)
