# Supplementary material for: A direct comparison of patient-reported outcomes and experiences in alternative models of maternity care in Queensland, Australia
Source: PLoS One. 2022 Jul 12;17(7):e0271105. doi: 10.1371/journal.pone.0271105 (PMC9275696; doi:10.1371/journal.pone.0271105)
Supplement: Attachment — Submitted filename: Response to Reviewers.docx [file pone.0271105.s006.docx]

PONE-D-22-04372

A direct comparison of patient-reported outcomes and experiences in alternative models of maternity care in Queensland, Australia.

| JOURNAL REQUIREMENTS | RESPONSE |
| --- | --- |
| 1. Please ensure that your manuscript meets PLOS ONE's style requirements, including those for file naming. The PLOS ONE style templates can be found at  [https://journals.plos.org/plosone/s/file?id=wjVg/PLOSOne_formatting_sample_main_body.pdf](https://urldefense.com/v3/__https:/journals.plos.org/plosone/s/file?id=wjVg*PLOSOne_formatting_sample_main_body.pdf__;Lw!!NVzLfOphnbDXSw!XfbPTQqRVQDOXUjPgK47DQZBbh8_fBA7V7VWoIFIfDg7r1LOXd1vHjf4IZ9OscICivqVvXc$) and  [https://journals.plos.org/plosone/s/file?id=ba62/PLOSOne_formatting_sample_title_authors_affiliations.pdf](https://urldefense.com/v3/__https:/journals.plos.org/plosone/s/file?id=ba62*PLOSOne_formatting_sample_title_authors_affiliations.pdf__;Lw!!NVzLfOphnbDXSw!XfbPTQqRVQDOXUjPgK47DQZBbh8_fBA7V7VWoIFIfDg7r1LOXd1vHjf4IZ9OscICcZpjKtM$) | Thank you. We have changed the size and formatting of headings, including table headings, to comply with PLOS ONE's style requirements. We also changed the indented text in tables to single tab indents to comply with formatting requirements in the manuscript and updated our file naming and formatting of Supplementary Material as per instructions on the PLOS ONE style template. |
| 2. Please provide additional details regarding participant consent. In the ethics statement in the Methods and online submission information, please ensure that you have specified what type you obtained (for instance, written or verbal, and if verbal, how it was documented and witnessed). If your study included minors, state whether you obtained consent from parents or guardians. If the need for consent was waived by the ethics committee, please include this information. | We have added the following detail regarding participant consent (Methods, lines 205-207):  *“Participation in the anonymous survey was assessed by the Ethical Review Committee as involving no more than minimal risk of harm and not requiring written consent. Return of the anonymous survey was taken as evidence of consent to participate.”* |
| 3. Thank you for stating the following financial disclosure:  "The authors received no specific funding for this work. The data on which this article is based was collected as part of a statewide survey program funded by the Queensland Government, at The University of Queensland. The funders nor the recipients of that funding had a role in study design, data collection and analysis, decision to publish, or preparation of the manuscript."  At this time, please address the following queries:  a) Please clarify the sources of funding (financial or material support) for your study. List the grants or organizations that supported your study, including funding received from your institution.  b) State what role the funders took in the study. If the funders had no role in your study, please state: “The funders had no role in study design, data collection and analysis, decision to publish, or preparation of the manuscript.” c) If any authors received a salary from any of your funders, please state which authors and which funders. d) If you did not receive any funding for this study, please state: “The authors received no specific funding for this work.”  Please include your amended statements within your cover letter; we will change the online submission form on your behalf. | Thank you for your guidance. Since no funding was received specifically for this study, please amend our financial disclosure to read:  *“The authors received no specific funding for this work.”* |
| 4. Thank you for stating the following in the Competing Interests section:  "We have read the journal's policy and the authors of this manuscript have the following competing interests: YM has previously received funding for the development of patient decision aids, including resources for women to choose between models of maternity care, and for establishing a state-wide survey of recent maternity consumers’ experience of maternity care across different models of care in Queensland, Australia. The funding bodies for that work had no involvement in the research reported here. EM is employed by a health service at which some participants in this study gave birth. EM was not employed by the health service at the time of data collection (2012) and the health service had no involvement or influence in the analysis of the data for the work reported in this manuscript. JT and ST have no competing interests to declare."  Please confirm that this does not alter your adherence to all PLOS ONE policies on sharing data and materials, by including the following statement: "This does not alter our adherence to  PLOS ONE policies on sharing data and materials.” (as detailed online in our guide for authors [http://journals.plos.org/plosone/s/competing-interests).](https://urldefense.com/v3/__http:/journals.plos.org/plosone/s/competing-interests).__;!!NVzLfOphnbDXSw!XfbPTQqRVQDOXUjPgK47DQZBbh8_fBA7V7VWoIFIfDg7r1LOXd1vHjf4IZ9OscICIPTXsso$)  If there are restrictions on sharing of data and/or materials, please state these. Please note that we cannot proceed with consideration of your article until this information has been declared.  Please include your updated Competing Interests statement in your cover letter; we will change the online submission form on your behalf. | Thanks for this further guidance. Please update our Competing Interests section to read:  “*We have read the journal's policy and the authors of this manuscript have the following competing interests: YM has previously received funding for the development of patient decision aids, including resources for women to choose between models of maternity care, and for establishing a state-wide survey of recent maternity consumers’ experience of maternity care across different models of care in Queensland, Australia. The funding bodies for that work had no involvement in the research reported here. EM is employed by a health service at which some participants in this study gave birth. EM was not employed by the health service at the time of data collection (2012) and the health service had no involvement or influence in the analysis of the data for the work reported in this manuscript. JT and ST have no competing interests to declare. This does not alter our adherence to PLOS ONE policies on sharing data and materials.”* |
| 5. We note you have included a table to which you do not refer in the text of your manuscript. Please ensure that you refer to Table 7 in your text; if accepted, production will need this reference to link the reader to the Table. | In-text references to Table 7 mistakenly referred to ‘Table 8’. This has been corrected. |
| 6. Please include a copy of Table 8 which you refer to in your text on page 37. | There is no Table 8. These in-text references were to Table 7. This has been corrected. |
| REVIEWER #1 COMMENTS |  |
| Abstracts have different wording. Please can they agree? The abstract in the paper is more informative. | Apologies for this inconsistency. The abstract in the online submission form has been updated to replicate the abstract in the paper. |
| Rationale for the study is well argued. The aim of this study was to directly compare women’s pregnancy, labour, birth, and postpartum outcomes and experiences across the major MMC categories offered in Queensland, Australia, using data collected in 2012 from a state-wide sample of women who had given birth in Queensland. This paper conducts secondary analyses of self-reported data collected in 2012 from a state-wide sample of women who had recently given birth in Queensland Australia. Please add to abstract where research is conducted and when. | The abstract now specifies that the data was collected in 2012 from a statewide sample of women who gave birth in Queensland, Australia. |
| This retrospective cohort study was conducted using data obtained from the 2012 Having a Baby in Queensland Survey. Please add response rate to abstract. | The response rate for the survey has been added to the Abstract. |
| This data is 10 years old. Please add to limitations and any relevant changes in policy or practice during this time. | Relevant practice and policy changes in the time since data collection are described in the Discussion in the context of specific findings, for example:  “*In 2020, the Queensland State Government Health Department released guidelines specifying the need to discuss and document the potential benefits and risks of recommended care and alternatives when a pregnant woman declines, or expresses an intention to decline, aspects of recommended maternity care [35]. The guidelines, for use in Queensland public hospitals where Standard Public Care is delivered, may have since affected the differences in informed consent processes between MMCs reported here, although the impact of implementing those guidelines on women’s experiences of informed consent has not been evaluated. Importantly, guidance on the informed decision-making processes which may precede women’s refusal of aspects of recommended maternity care is not provided. Further research is needed to confirm whether the differences between MMCs in Queensland reported here have been sustained since the time of data collection.”*  We have also added the following description of relevant changes to policy to our Discussion, particularly in light of our limitation associated with excluding comparisons with Private Midwifery Care:  *At the time of data collection in 2012, significant Australian Federal government reforms to improve access to Private Midwifery Care had recently been enacted. In 2010, Australia introduced public healthcare funding for care delivered by Medicare eligible Private Practice Midwives, which included Medicare rebates for antenatal and postpartum care delivered by eligible Private Practice Midwives alongside mechanisms for collaborative arrangements between eligible Private Practice Midwives and hospitals to provide ‘visiting access’ for in-hospital intrapartum care with continuity of care from the woman’s chosen private midwife. Queensland, where the current study was conducted, utilised the national reforms to provide visiting access to Medicare eligible private practice midwives “earlier and more fully” than other Australian states and territories [54]. The most recent data suggest that Private Midwifery Models account for 2.2% of all MMCs available in Australia and 5% of all MMCs in Queensland [55], although how this compares to earlier pre-reform availability and the proportion of women who use Private Midwifery Care remains unknown. Intrapartum care at home (i.e., homebirth) has remained un-funded in the national (public) health insurance system (i.e., all costs are privately funded by the woman) [56] and the percentage of women who give birth at home in Queensland has only marginally increased from 0.1% to 0.2% in the period between 2012 and 2019 [46].* |
| Of 2,802 women, 18.2% received Standard Public Care, 21.7% received GP Shared Care, 12.9% received Public Midwifery Continuity Care, and 47.1% received Private Obstetric Care. Please specify differences between these models. | The differences between models are outlined in Table 1 and Table 2. We have added further detail to the table to specify continuity of a named care provider and the determination of specific hospitals available for intrapartum care based on visiting rights of the named care provider in private models.  We further discuss specific differences between models in the context of specific findings, for example:  “*Continuity of carer and having a known care provider during labour and birth were more likely in Public Midwifery Continuity and Private Obstetric models. This was unsurprising, given that both models are structured for women to receive care from the same midwife, team of midwives or named obstetrician across antepartum and intrapartum care”* (lines 712-716). |
| Logistic regression was used to estimate the odds of 34 outcomes and experiences associated with three models (GP Shared Care, Public Midwifery Continuity Care, Private Obstetric Care) compared with Standard Public Care, adjusting for relevant maternal characteristics and clinical covariates. Line 73. Does private midwifery care provide continuity? | We have added detail to Table 1 specifying that continuity of care from a chosen care provider is provided for both Private Midwifery and Private Obstetric Care. |
| Line 93 Specify which antenatal care providers ? | We have specified that this evidence refers to women’s experience with their GPs with the addition:  *“women are generally not informed about Public Midwifery Continuity Care by their by their GPs, who are responsible for the majority of referrals to a specific MMC”* |
| Table 1 clarify who provides obstetric care if required in each model and who pays for this? | Specialist obstetric care is provided consistent with the funding terms outlined in Table 1. Where specialist obstetric care is not available within that model (i.e., Private Midwifery Care), women can access obstetric care via an alternative model with the funding terms of the model they access for specialist obstetric care. We have made revisions to Table 1 to reflect this. |
| Line 239 Classification of MMC did not capture transitions between models of care during pregnancy. Where women transitioned between models of care during the index pregnancy, women were classified into the final model received in pregnancy to allow comparison of outcomes under a single model of care received. My understanding is that women are not classified by the model they started with, but the model they ended up in. Is this correct? If so, please justify as this is not intention to treat analysis and introduces bias? | Yes, this is correct. We attempted to adjust for the effects of transitions associated with maternal characteristics and clinical risk as much as possible with available data, but agree that the unavoidable bias, especially for women’s early antenatal MMC decision making, is important.  We have added to following to our Discussion:  “*However, our algorithm for categorising women according to a specific MMC did not account for transitions between models (since women were classified as having received a single MMC) and was biased towards classification of the final model received (i.e., for intrapartum care). Importantly for decision-making about MMC allocation and referral in early pregnancy, this means our analyses is based on ‘treatment received’ rather than ‘intention-to-treat’. We attempted to reduce this bias as much as possible by adjusting analyses for clinical characteristics that may affect transitions between models from initial allocation to time of birth.”* |
| Line 304 Low response rate. Comment upon limitations of study. Please add weblink to survey questions if available. | We have now discussed the low response rate and generalisability of the sample in our Discussion (lines 773-789).  The specific survey questions are available in the report from the survey (new ref #28) which is available online. The web link to this source is now provided with the reference. The measurement of variables used in the analyses described in the paper is further detailed in Supplementary Materials (S1 Table). |
| There are many many comparisons in this paper by differing models. Is it possible to produce a diagram/infographic summarising these to help the reader interpret the findings. | Thank you for this suggestion. We think it adds a lot to the usefulness of how we report on the findings. We have added a graphical summary of the indicators that have a significant association with at least one MMC compared to Standard Public Care (see additional Figures 1 and 2). |
| Line 618 Please report key infant outcomes here for all models as a balancing measure. | We have added the following paragraph about findings for significant infant outcomes to our Discussion:  *“We found comparably decreased odds of NICU admission for both Public Midwifery Continuity Models and Private Obstetric Models compared to Standard Public Care, and decreased odds of preterm birth for Public Midwifery Continuity Care, even after accounting for relevant maternal and clinical characteristics. Trials conducted internationally have found similar (though smaller on average) reduced likelihood of preterm birth for women who receive Midwifery Continuity Care than those in other MMCs [16]. NICU admission has been found to be lower in a Public Midwifery Continuity Model than in either Standard Public or Private Obstetric Care for women receiving care in one hospital in Australia [19], but not significantly different between Midwifery-led care and other models in an international review and meta-analysis [17]. Notably, we were unable to compare MMCs on infant survival outcomes in this study because our sampling strategy excluded women who had a stillbirth or a neonatal death from our standard data collection procedures, although lower risk of fetal and infant loss have been found for Midwifery Continuity Care compared to other MMCs in the international literature [16].”* |
| There is little reference to work in the filed in the discussion. What does this work add to this body of international literature? | The major contributions of this paper to the international literature include the comparison of multiple distinct models of care and on the basis of experiential measures, with previously unmatched breadth.  We have added more information on the international literature to our Introduction, since it highlights similar gaps in evidence to the Australian literature that our contribution attempts to address. Additions include:  “*Consistent with the Australian literature comparing MMCs [5] comparative studies conducted internationally have primarily focused on comparing midwifery-led care with other models on clinical outcomes [15–17]. A recent systemic review and meta-analysis of studies comparing midwifery-led care with standard or usual care found lower odds of caesarean births and episiotomy for women in midwifery-led models [17]. An earlier systematic review and meta-analysis comparing midwifery continuity care with other models of care (examined as one comparison model comprising Obstetrician-led, GP Shared Care, or Standard Care) internationally indicated that women in midwifery-led models were less likely to have an epidural during labour, an assisted vaginal birth and an episiotomy and more likely to experience an unassisted vaginal birth, regardless of their pregnancy risk or if they received caseload versus team midwifery care [16]. Women in midwifery-led models were also more likely to demonstrate a higher level of satisfaction with care compared to other models [16].”*  “*An international systematic review and meta-analysis found insufficient studies comparing MMCs on the basis of women’s experiences of care to warrant their inclusion in synthesising effects [17].”*  “*There remains a paucity of studies comparing the various MMCs available to women beyond the comparison of only two models for useful informed MMC decision-making at both an Australian and international level.”*  We have also referred to specific international literature in our Discussion in the context of our findings and limitations of our research, including:  *“A further limitation associated with our MMC coding algorithm was failing to distinguish between Caseload and other Midwifery Continuity models, which have been found to differ in clinical outcomes in other settings (e.g., [42]).”*  and also:  *“We found comparably decreased odds of NICU admission for both Public Midwifery Continuity Models and Private Obstetric Models compared to Standard Public Care, and decreased odds of preterm birth for Public Midwifery Continuity Care, even after accounting for relevant maternal and clinical characteristics. Trials conducted internationally have found similar (though smaller on average) reduced likelihood of preterm birth for women who receive Midwifery Continuity Care than those in other MMCs [16]. NICU admission has been found to be lower in a Public Midwifery Continuity Model than in either Standard Public or Private Obstetric Care for women receiving care in one hospital in Australia [19], but not significantly different between Midwifery-led care and other models in an international review and meta-analysis [17]. Notably, we were unable to compare MMCs on infant survival outcomes in this study because our sampling strategy excluded women who had a stillbirth or a neonatal death from our standard data collection procedures, although lower risk of fetal and infant loss have been found for Midwifery Continuity Care compared to other MMCs in the international literature [16].”* |
| REVIEWER #2 COMMENTS |  |
| Thank you for the opportunity to review this paper. I found the paper to be very well written and thorough. I preferred the tables in the supplementary that included p values and think this format would be better for the wider readership than the tables in the manuscript that only have OR presented as it helps the reader focus on the statistically significant outcomes. There is also little reference to the CI in the results but the tables include them. | Thanks for your positive feedback and the suggestion to include p-values in the tables within the manuscript. Unlike the tables in Supplementary material, the tables within the paper present the results from multiple models (with different adjustments). The significance is denoted using * for *p* <.01; and ** for *p* <.001. To include the raw p-values alongside the data presented would require twice the number of tables. On balance, we feel that the readability is better with fewer tables that combine the findings from equivalent analyses with varying adjustments and indicating the significance of associations using the two standard (but conservative) thresholds rather than the p-values themselves. |
| My biggest concern is with the age of the dataset being over 10 years since collection. You haven't mentioned in the limitations the fact that the data is over 10 years old and that there has been development of the PPM and MGP model across Australia and in QLD during that time. When you collated this data the 2010 maternity care changes had just come into effect and there would have been very few endorsed (eligible) midwives at the time, now there are many more and now also with visiting rights, especially in QLD. I think this really needs to be explained in this paper. Otherwise you are suggesting this data is more current and relevant than it is. | Thank you for this suggestion. We agree, and have now added the following to our Discussion:  *“At the time of data collection in 2012, significant Australian Federal government reforms to improve access to Private Midwifery Care had recently been enacted. In 2010, Australia introduced public healthcare funding for care delivered by Medicare eligible Private Practice Midwives, which included Medicare rebates for antenatal and postpartum care delivered by eligible Private Practice Midwives alongside mechanisms for collaborative arrangements between eligible Private Practice Midwives and hospitals to provide ‘visiting access’ for in-hospital intrapartum care with continuity of care from the woman’s chosen private midwife. Queensland, where the current study was conducted, utilised the national reforms to provide visiting access to Medicare eligible private practice midwives “earlier and more fully” than other Australian states and territories [54]. The most recent data suggest that Private Midwifery Models account for 2.2% of all MMCs available in Australia and 5% of all MMCs in Queensland [55], although how this compares to earlier pre-reform availability and the proportion of women who use Private Midwifery Care remains unknown. Intrapartum care at home (i.e., homebirth) has remained un-funded in the national (public) health insurance system (i.e., all costs are privately funded by the woman) [56] and the percentage of women who give birth at home in Queensland has only marginally increased from 0.1% to 0.2% in the period between 2012 and 2019 [46].“*  We have also added this point to the Discussion to highlight the potential importance of distinguishing between caseload and other midwifery continuity models:  *A further limitation associated with our MMC coding algorithm was failing to distinguish between Caseload and other Midwifery Continuity models, which have been found to differ in clinical outcomes in other settings (e.g., [42]).* |
